# Determining cancer stage at diagnosis in population-based cancer registries: A rapid scoping review

**DOI:** 10.3389/frhs.2023.1039266

**Published:** 2023-03-08

**Authors:** Li Pung, Rachael Moorin, Richard Trevithick, Karen Taylor, Kevin Chai, Cristiana Garcia Gewerc, Ninh Ha, Stephanie Smith

**Affiliations:** ^1^ School of Population Health, Curtin University, Perth, WA, Australia; ^2^ Public Health, North Metropolitan Health Service, Perth, WA, Australia; ^3^ School of Population and Global Health, The University of Western Australia, Perth, WA, Australia; ^4^ Department of Health, Western Australia Cancer Registry, Clinical Excellence Division, Perth, WA, Australia; ^5^ Cancer Network WA, North Metropolitan Health Service, Perth, WA, Australia

**Keywords:** rapid scoping review, registry-derived stage, population-based cancer stage, population-based cancer registries, cancer stage, stage at diagnosis

## Abstract

**Introduction:**

Population-based cancer registries are the main source of data for population-level analysis of cancer stage at diagnosis. This data enables analysis of cancer burden by stage, evaluation of screening programs and provides insight into differences in cancer outcomes. The lack of standardised collection of cancer staging in Australia is well recognised and is not routinely collected within the Western Australia Cancer Registry. This review aimed to explore how cancer stage at diagnosis is determined in population-based cancer registries.

**Methods:**

This review was guided by the Joanna-Briggs Institute methodology. A systematic search of peer-reviewed research studies and grey literature from 2000 to 2021 was conducted in December 2021. Literature was included if peer-reviewed articles or grey literature sources used population-based cancer stage at diagnosis, and were published in English between 2000 and 2021. Literature was excluded if they were reviews or only the abstract was available. Database results were screened by title and abstract using Research Screener. Full-texts were screened using Rayyan. Included literature were analysed using thematic analysis and managed through NVivo.

**Results:**

The findings of the 23 included articles published between 2002 and 2021 consisted of two themes. (1) “Data sources and collection processes” outlines the data sources used, as well as the processes and timing of data collection utilised by population-based cancer registries. (2) “Staging classification systems” reveals the staging classification systems employed or developed for population-based cancer staging, including the American Joint Committee on Cancer's Tumour Node Metastasis and related systems; simplified systems classified into localised, regional, and distant categories; and miscellaneous systems.

**Conclusions:**

Differences in approaches used to determine population-based cancer stage at diagnosis challenge attempts to make interjurisdictional and international comparisons. Barriers to collecting population-based stage at diagnosis include resource availability, infrastructure differences, methodological complexity, interest variations, and differences in population-based roles and emphases. Even within countries, disparate funding sources and funder interests can challenge the uniformity of population-based cancer registry staging practices. International guidelines to guide cancer registries in collecting population-based cancer stage is needed. A tiered framework of standardising collection is recommended. The results will inform integrating population-based cancer staging into the Western Australian Cancer Registry.

## 1. Introduction

Cancer staging at an individual level is integral to determining clinical management and estimating prognosis. Population-level cancer stage at diagnosis, defined as the extent to which a cancer has spread at initial diagnosis, is used to analyse the burden of cancer by stage and associated trends over time, which assists with planning for predicted demand on cancer health services and enables the evaluation of outcomes from cancer screening programs (1–3). Population-based cancer stage analysis also assists in understanding differences in cancer outcomes and survival (3, 4). While international comparisons showed differences in overall cancer survival between countries, the differences are mainly explained by stage at diagnosis and stage-specific survival variations (4). Therefore, completeness, consistency and comparability of staging classification are required to enable international comparisons of cancer outcomes (5–7).

The American Joint Commission on Cancer's (AJCC) Tumour Node Metastases (TNM) is a comprehensive cancer staging classification system most widely used globally for staging solid tumours. However, it is not always available to population-based cancer registries (PBCRs) nor collected routinely by them (5, 8). Limitations in collecting stage data by PBCRs generally relate to the availability and completeness of recorded cancer stage and other stage information in routinely received or accessible data sources (7). Furthermore, the collection of stage at diagnosis requires significant resources, which poses a substantial challenge for PBCRs that are resource constrained.

In the face of these barriers, PBCRs use different approaches to collect population-based cancer stage at diagnosis. This includes the simplified staging systems developed specifically for use by PBCRs for population-level analyses, including the Surveillance, Epidemiology and End Results' (SEER) Summary Stage and the European Network of Cancer Registries' (ENCR) Condensed TNM staging system (7, 9, 10). As a result, different PBCRs may use different staging classification systems to collect stage at diagnosis at a population-level. If there are no validated means of converting between different staging classification systems, then this can result in difficulties in making comparisons of stage at diagnosis data between PBCRs.

The lack of standardised collection of staging data in Australia has been identified as a major gap in the National Cancer Data Strategy by Cancer Australia (3). This led to the national Stage, Treatment and Recurrence project (STaR project, also known as National Collection of Registry-Derived Stage project) which enabled the consistent collection of Registry-Derived stage for the five highest incidence tumour groups (prostate, breast, lung, colorectal, and melanoma) across all Australian PBCRs for cancer cases diagnosed in 2011 (1, 11). Registry-Derived (RD) stage was defined as the best estimate of cancer stage at diagnosis used for population-based analysis, as determined by PBCRs from available data sources (12). The Western Australian Cancer Registry (WACR) is one such PBCR which was involved in the STaR project.

The WACR was developed in 1982 and has since provided population-based cancer data for use in the planning of health care services and the support of cancer-related research at local, national, and international levels (13). Except for the duration of the STaR project, from 2018 onwards the WACR has collected pathological cancer stage at diagnosis incidentally when identified during routine cancer registration processes, however it is not a routinely collected data item. In alignment with Cancer Australia's position on the requirement for improved stage data collection, the Western Australia (WA) Cancer 2020–2025 Implementation Plan includes the key strategic action to “Develop a timely data collection for cancer stage at diagnosis” (14). Subsequently, the WA Cancer Staging Project has commenced which aims to develop, deliver and evaluate a state-wide population-based staging approach within the WACR.

A key consideration for the project has been how to ensure comparability and consistency with PBCRs from other Australian jurisdictions and from other countries, with the aim of enabling interjurisdictional and international benchmarking. At the time of this rapid scoping review, there was limited literature existing that examined how PBCRs collect population-based cancer stage at diagnosis. The aim of this rapid scoping review was to explore how population-based cancer stage at diagnosis is determined at a population-level. By providing an overview of the literature on population-based cancer staging approaches used by PBCRs, the findings of this review will provide critical information to inform the development and implementation of population-based cancer staging in the WACR.

## 2. Methods

A rapid scoping review was identified as the most appropriate approach as scoping reviews provide a broader overview of the literature compared to systematic reviews that address a targeted question (15) and uses a more systematic approach compared to literature reviews (16). The study was designed to be conducted in three months to present preliminary findings to key stakeholders of the WA Cancer Staging Project. Therefore, a rapid scoping review was conducted as defined by Tricco et al. (17) whereby aspects of the review process are simplified or omitted to enable the production of information in a timely manner. In this rapid review, the screening process was simplified where only a portion of articles for title and abstract screening were reviewed by a second reviewer and full-text screening was conducted by one reviewer. Yet, any queries regarding inclusion of the full-text were discussed with the second reviewer.

The review was undertaken following the Joanna-Briggs Institute methodology for scoping reviews (18–20). The Cochrane Database for Systematic Reviews (21) and the Joanna Briggs Institute (JBI) Evidence-based Practice Database (22) were searched and no existing reviews in this area were identified. The research team involved expertise in the WACR, PBCRs, population health, health psychology, cancer nursing, data science and qualitative, quantitative and mixed methods research. The composition of the team created a diverse range of perspectives on reviewing the data.

Registration of the protocol with Figshare (23) and Open Science Framework (24) were explored. Whilst protocol registration is not currently a requirement for scoping reviews as per the JBI framework (18) the timeframe to register was not a possibility due to the project timeframe and the need to conduct the review within three months to present the preliminary findings to the WA Cancer Staging Project key stakeholders.

As per the JBI framework, an initial protocol was compiled prior to undertaking the review (18). The protocol outlined the background and gaps in the literature, aims, methods and reporting of the review and was peer-reviewed by an experienced health researcher with expertise in scoping review methodology and qualitative methods. The initial protocol was positively received. The protocol was amended when the research team decided to conduct a rapid scoping review instead. The priori protocol was available to all authors and assisted the systematic search and screening process.

### 2.1. Eligibility criteria

Literature was included if the following inclusion criteria were met:

a) Peer-reviewed articles or grey literature sources using population-based cancer stage at diagnosis
b) Published in English
c) Published from year 2000 to 2021 inclusive

Literature was excluded based on the following exclusion criteria:

a) Only the abstract was available
b) Reviews

The timeframe was chosen due to the emergence of registry staging during this period based on discussions between all authors. One of the authors was familiar with the literature in this area and guided the timeframe and grey literature sources to search.

### 2.2. Search strategy

The three-step search strategy as recommended by JBI (18) was adapted for the literature search. The first step involved the initial limited search of MEDLINE in consultation with an experienced research librarian using the research question and preliminary key terms. In consultation with the authors and the research librarian, the search terms were compiled, and specific research databases were chosen (MEDLINE, Embase, CINAHL, and Web of Science). The second step involved the full search of the final search terms in the selected research databases in December 2021. Table 1 lists the search terms used developed in consultation with LP, SS, RT, and RM and the research librarian. Google Scholar search terms used were simplified to fit within the character limit for searches. All searches were limited to English and between the years 2000–2021. The third step occurred after screening of the articles was completed and involved searching the reference lists of papers that met the inclusion criteria.

**Table 1:** Search terms used for literature search.

Keywords	Alternative keywords	Database search terms combined with the Boolean operator AND (MEDLINE, Embase, CINAHL, Web of Science)	Google Advanced Search search terms combined with Boolean operator AND	Google Scholar search terms
Registry derived stage Cancer registry	Registry derived stage, RD stage, population-based cancer stage, registry stage, stage data collection, registry-based stage, cancer registry, tumour registry, tumor registry	“Registry derived stag*” OR “Population based cancer stag*” OR “RD stag*” OR “Registr* stag*” OR “Stag* data collect*” OR “Registr* based stag*” OR “Cancer registr*” OR “Tumour registr*” OR “Tumor registr*”	(Registry derived OR RD OR population based OR registry OR registry based) (stage OR stages OR staging) OR “stage data collection” OR (cancer OR tumour OR tumor) (registry OR registries)	((Registry derived OR population based OR registry OR registry based) (stage OR stages OR staging))
Cancer stage	Neoplasm staging, cancer staging, tumour staging, tumor staging, stage at diagnosis, TNM stage, pathology stage	“Neoplasm stag*” OR “Cancer stag*” OR “Tumour stag*” OR “Tumor stag*” OR “Stag* at diagnos*” OR “TNM stag*” OR “Patholog* stag*”	Neoplasm OR cancer OR tumour OR tumor OR TNM OR pathological OR pathology) (stage OR staging OR stages) OR “stage at diagnosis”	((Business OR coding OR stage OR staging) (rule OR rules) OR (cancer OR medical OR clinical OR pathology) (record OR records OR report OR reports))
Business rules Pathology reports	Business rules, coding rules, cancer records, medical records, clinical records, pathology report, staging rules	“Business rule*” OR “Coding rule*” OR “Cancer record*” OR “Medical record*” OR “Clinical record*” OR “Pathology report*” OR “Stag* rule*”	(Business OR coding OR stage OR staging) (rule OR rules) OR (cancer OR medical OR clinical OR pathology) (record OR records OR report OR reports)	

Searches for grey literature defined as including reports, theses, conference proceedings, technical specifications and standards, translations, bibliographies, technical and commercial documentation, and official documents (25), were also undertaken. The grey literature search involved conducting searches in Google Scholar and Google advanced search, where the first 200 results, as recommended by Haddaway et al. (26), were reviewed by the first reviewer. Official websites of population-based cancer registries and organisations as identified from discussion with other authors were also searched for grey literature, including the websites for SEER (27), National Cancer Registration and Analysis Service (NCRAS) (28), Canadian Partnership Against Cancer (29), the International Cancer Benchmarking Partnership (ICBP) (30), ENCR (31), and International Agency for Research on Cancer (IARC) (32).

### 2.3. Selection of sources of evidence

The search results were imported into Research Screener which is a semi-automated machine learning tool developed at Curtin University (33). Research Screener facilitates title and abstract screening and automatically removes duplicate articles, enabling more rapid screening of literature for reviews (33) as was required for the short timeframe for the review. Seed articles are defined as articles that have been identified by the researchers as highly relevant based on their eligibility criteria and are used by the Research Screener algorithm to initially rank articles according to relevance (33). Six seed articles (5–7, 11, 34, 35) were chosen for use with Research Screener in consultation with the research team. The algorithm repeatedly re-ranks the remaining unread articles based on what articles the reviewer has included and excluded.

The first reviewer screened all articles identified from the search of research databases by title and abstract using the eligibility criteria. A second reviewer independently screened the first 50 articles produced in Research Screener by title and abstract for eligibility and subsequently screened any additional articles identified by the first reviewer as meeting the eligibility criteria. Uncertainty or disagreements were resolved through discussion between the two reviewers. The eligible full-texts were imported into Rayyan's Systematic Review Screening Software (36) and the first reviewer performed the full-text review of eligible articles. Rayyan is a tool that assists with facilitating the systematic search and selection of studies for reviews, with features that demonstrate when multiple reviewers agree or disagree on the inclusion and exclusion of articles, and allowing the reason for exclusions to be listed per excluded article (36). Two texts resulted in uncertainty to include and were resolved by discussion between four of the authors (LP, SS, RT, RM). The reference lists of articles that met the eligibility criteria during full-text review were checked to identify additional relevant articles.

### 2.4. Data extraction

Data was extracted utilising a charting table developed by the two reviewers and adapted from the JBI template source of evidence details, characteristics and results extraction instrument (18). The table included the author(s), year of publication, country of origin, aims, type of article, and key findings that relate to the scoping review question.

### 2.5. Synthesis of results

Included full-texts were imported to qualitative data analysis software NVivo (37) which was used to manage the data. Thematic analysis was used to compare and contrast the findings across studies to enable identification and interpretation of patterns within the data (38). The data analysis was guided by Braun and Clarke's (38) six stages of thematic analysis (familiarisation with the data, generating initial codes, searching for themes, reviewing themes, defining and naming themes, producing the report) using an inductive and iterative process. The first reviewer read and reread all the included texts to familiarise with the data noting down preliminary ideas, generated a list of initial codes from the entire dataset in a data-driven approach, and grouped common codes to form themes. The themes at the level of the included codes and the overall dataset using a thematic map were reviewed by the first and second reviewers. The subsequent themes and subthemes were reviewed by all authors. Themes were further analysed and defined by considering how they conveyed a narrative related to the research question. Lastly, the results of the data analysis were written up into this article.

## 3. Results

**Figure 1:**
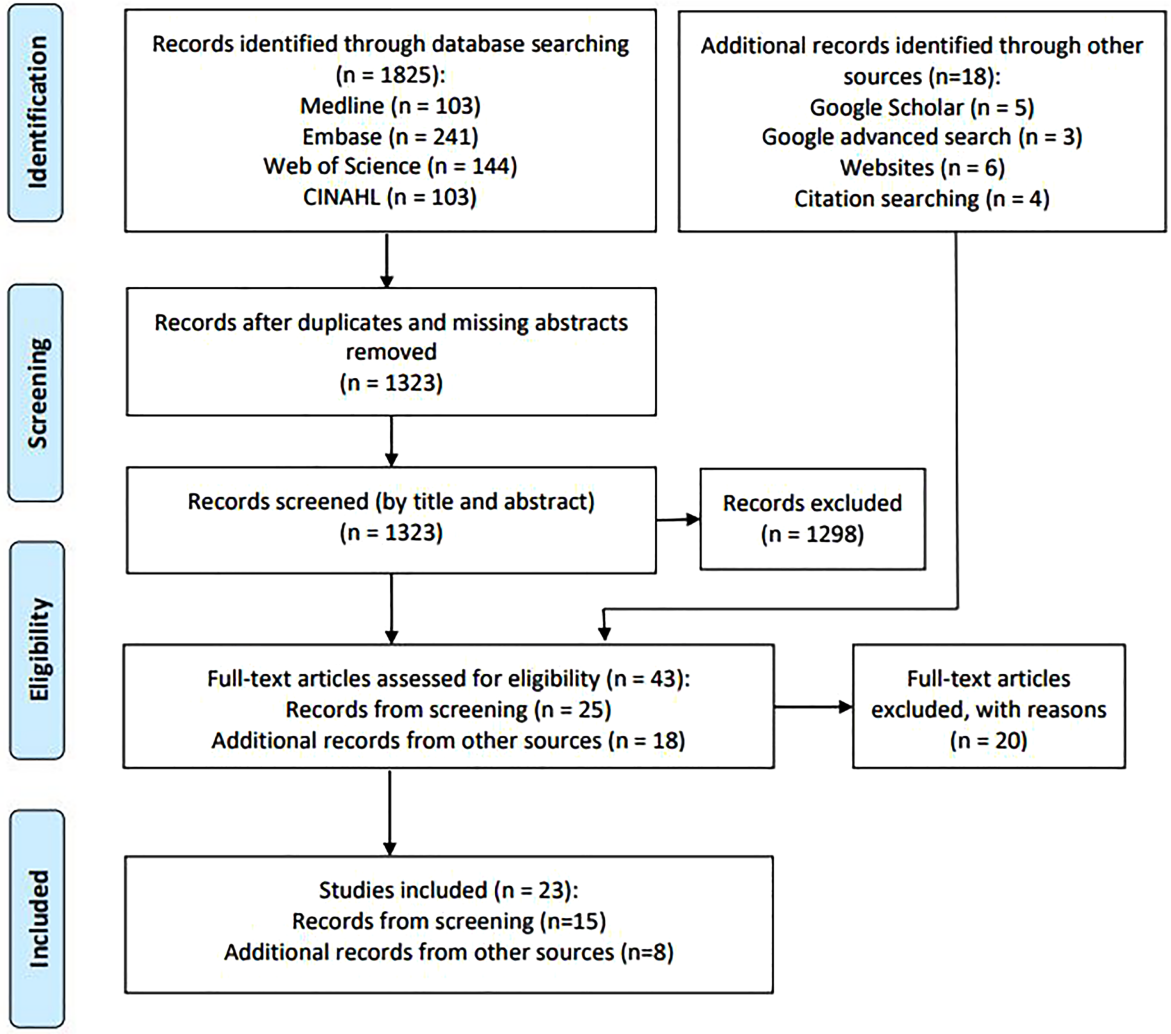
PRISMA-ScR flow diagram for the rapid scoping review.

The search identified a total of 1,843 records (*n* = 1,825 from database searches, *n* = 18 from other sources). After removal of duplicates and records missing abstracts, the remaining 1,323 records from database searches were screened by title and abstract. The resultant records as well as records from other sources were assessed for eligibility (*n* = 43) (Figure 1). The results of the search and the study inclusion process are presented in the adapted PRISMA-ScR flow diagram (Figure 1) (19). The PRISMA-ScR flow diagram was adapted as the records from additional sources did not consistently have an abstract and as a result could not be screened by title and abstract using Research Screener. Instead, these records moved straight to the full-text review stage.

The final dataset consisted of 23 texts, including 15 peer-reviewed journal articles and eight electronic documents from official websites of cancer registries and cancer organisations. All texts were published between 2002 and 2021 (Table 2).

**Table 2:** Characteristics of included sources of evidence.

References	Year	Country	Type of document	Aim/Purpose	Staging Classification System	Data sources
Adamo et al. (39)	2022	USA	Staging Manual/Rules	Explains data item descriptions, codes, and coding instructions for SEER cancer registry for cases diagnosed for the year 2022 onwards	SEER Summary Stage SEER Extent of Disease	N/A
Aitken et al. (40)	2018	Australia	Quantitative	Assessment of the feasibility of implementing the Toronto Guidelines within a national population cancer registry	Toronto Paediatric Cancer Stage Guidelines	Computer algorithms Manual staging
Aitken et al. (41)	2016	Australia	Staging Manual/Rules	Provides detailed staging criteria for Toronto Paediatric Cancer Stage Guidelines	Toronto Paediatric Cancer Stage Guidelines	N/A
Benitez-Majano et al. (6)	2016	England	Quantitative	Development of an algorithm to determine stage at diagnosis using multiple population-based data sources	UICC TNM	Clinical audit data
Berrino et al. (10)	2002	England France Italy Sweden USA	Staging Manual/Rules	European Network of Cancer Registries’ guide for using Condensed TNM for cancer staging	Condensed TNM	N/A
Bryan et al. (42)	2018	Canada	Quantitative	Presents national data on cancer incidence by stage at diagnosis for Canada	Collaborative Stage System	N/A
Cabasag et al. (43)	2021	Canada England France USA	Quantitative	International comparisons of stage information available, completeness, and misclassification from stage conversion algorithms developed	TNM SEER Summary Stage Extent of Disease	Timing of data collection 120 days Hospital data Death certificates Autopsy reports
Collaborative Staging Task Force of the American Joint Committee on Cancer (44)	2007	Canada USA	Staging Manual/Rules	Guide on what Collaborative Stage System is and how to use it	Collaborative Stage	Computer algorithms Manual staging Timing of data collection 120 days
Cunningham et al. (45)	2008	New Zealand	Quantitative	Audit assessing the reliability of New Zealand Cancer Registry colon cancer data	SEER Summary Stage	Manual staging Death certificates Pathology reports Coroner's reports Hospital discharge reports (public, private)
Gupta et al. (46)	2016	Argentina Australia Canada Colombia El Salvador England France Guatemala India Indonesia Italy Malawi Philippines South Korea Switzerland USA	Qualitative	Development of guidelines for the population-based cancer staging of paediatric cancers	Toronto Paediatric Cancer Stage Guidelines	Pathology reports Hospital discharge abstracts Death certificates
Henson et al. (47)	2020	England	Quantitative	Guide on the data sources comprising the national cancer registration dataset in England	TNM	Manual staging Multidisciplinary team meeting software Molecular testing results Imaging systems Clinical audit data Chemotherapy e-prescribing systems Imaging systems Pathology reports Post-mortems Treatment records Hospital activity records Hospital Patient Administration Systems
Lawrance et al. (11)	2019	Australia	Quantitative	Comparison of the improvements in completeness and accuracy of stage data between registry derived stage and degree of spread	AJCC TNM Registry-derived stage Degree of Spread	Manual staging Timing of data collection 120 days Pathology reports Electronic hospital notifications Clinical information
Meng et al. (34)	2020	Australia	Quantitative	Comparison of stage data quality and completeness between registry-derived stage, pathology stage linked with hospital metastasis codes, and South Australian Clinical Cancer Registry Stage	Registry-derived stage AJCC TNM	Manual staging Pathology Hospital admission Deaths from the Births, Deaths and Marriages registry Radiotherapy notifications
Minicozzi et al. (48)	2017	Bulgaria Catalan Estonia Ireland Italy Netherlands Spain	Quantitative	Analysis of the quality of stage data received by cancer registries participating in the EUROCARE-5 study	TNM Condensed TNM Extent of Disease	N/A
Noone et al. (49)	2015	USA	Quantitative	Examination of how frequently T, N and M were missing in medical records and required registrars to directly assign stage	AJCC TNM	Manual staging
Piñeros et al. (7)	2019	Australia Canada England France India Switzerland USA Zimbabwe	Qualitative	Development of Essential TNM staging system and explanation on how to use it	Essential TNM	N/A
Public Health England (50)	2020	England	Staging Manual/Rules	Guide on what data the National Cancer Registration and Analysis Service receives	Not stated	Manual staging Somatic molecular data Germline molecular data Systemic Anti-Cancer Therapy dataset Radiotherapy dataset Cancer Outcomes and Service Dataset National Cancer Diagnosis Audit National Clinical Audits for Lung, Breast, and Prostate Cancer Hospital Episodes Statistics Diagnostic imaging dataset Prescription data National Cancer Waiting Times Monitoring data National Cancer Patient Experience Survey Patient Reported Outcome Measures datasets National Head and Neck Cancer Audit
Ruhl et al. (51)	2021	USA	Staging Manual/Rules	Coding instructions on how to determine SEER Extent of Disease 2018	SEER Extent of Disease 2018	Timing of data collection 4 months
Ruhl et al. (9)	2021	USA	Staging Manual/Rules	Coding instructions on how to determine SEER Summary Stage 2018	SEER Summary Stage 2018	N/A
Siesling et al. (52)	2013	Ireland Italy Northern The Netherlands	Quantitative	Examines whether cancer registries from the European Cancer Health Indicators Project collect indicators stage at diagnosis, cancer treatment delay, and compliance with cancer guidelines	TNM Others not stated	Pathology laboratories Hospital oncology records Other hospital records Radiotherapy departments Haematology laboratories Death certiﬁcates
Stevens et al. (53)	2008	New Zealand	Quantitative	Examines the completeness and accuracy of lung cancer data in the New Zealand Cancer Registry	Extent of Disease	Timing of data collection 4 months Laboratory reports Discharge summaries from public and private hospitals Death certificates Autopsy reports
Threlfall et al. (35)	2005	Australia	Quantitative	Determine the feasibility of routinely collecting stage data in the WA Cancer Registry	TNM	Manual staging Pathology reports Hospital medical records
Walters et al. (5)	2013	Canada England	Quantitative	Comparison of stage at diagnosis from six countries in the ICBP through developed stage conversion algorithms	TNM Duke's FIGO Extent of Disease	N/A

Two key themes were identified. (1) “Data sources collection processes,” reviews the data sources used and processes, including the timing of data collection. (2) “Staging classification systems,” outlines the various classification systems including tumour, node and metastasis; those that are categorised into local, regional, and distant; and miscellaneous staging classification systems. Table 3 summarises the key themes and subthemes.

**Table 3:** Key themes and subthemes.

Themes	Subthemes
Data sources and collection processes	Data sources used Processes and timing of data collection
Staging classification systems	Based on tumour, node, and metastasis Categorised into local, regional, and distant Miscellaneous staging classification systems

### 3.1. Theme 1: Data sources and collection processes

This theme covers the data sources employed and the data collection processes utilised by PBCRs, including whether data was processed by manual review or using computer algorithms and the timeframe to collect cancer information to determine population-level cancer stage at diagnosis.

#### 3.1.1. Data sources used

PBCRs from different countries used different data sources for population-based cancer staging. The frequently used data sources were pathology reports (11, 34, 45–47, 52, 53), hospital notifications and discharge summaries (11, 34, 45–47, 53), death certificates (34, 43, 45, 46, 52, 53) and autopsy reports (43, 45–47, 53), and medical records (11, 47, 52). Under national and jurisdictional legislation, PBCRs in Australia are required to be notified of cancer diagnoses (11, 34, 35) and routinely received notification sources were pathology reports, hospital morbidity data, and death certificates (11, 34, 35). Other sources available varied depending on the jurisdiction. For example, the South Australian Cancer Registry received radiotherapy notifications (34). Similarly, the New Zealand Cancer Registry received pathology reports with new diagnoses of cancer under the Cancer Registry Act 1993, as well as hospital discharge reports, death certificates, and autopsy reports (53). The most routinely used data sources for the European PBCRs were pathology laboratories (used by 100% of PBCRs), hospital oncology records (93%), other hospital records (97%), radiotherapy departments (83%), haematology laboratories (80%), and death certificates (78%) (52).

Chemotherapy systems, radiotherapy systems, imaging systems, multidisciplinary team (MDT) meeting data, molecular testing results, and clinical audit data were less commonly available data sources for PBCRs. The NCRAS in England was the single PBCR that reported access to those aforementioned sources (6, 47, 50). The NCRAS also had additional access to multiple different linked datasets for its cancer registration processes (6, 47, 50), including national data on hospital activity, patient waiting times, diagnostic imaging, cancer screening, mortality, and national cancer audits (47, 50). The National Cancer Patient Experience Survey and the Patient Reported Outcome Measures datasets were additional accessible data sources that were linked to cancer registration data (50).

Though pathology reports were a commonly utilised data source for PBCRs, several limitations were identified regarding their use as a source of stage data (43, 52). First, pathological stage was not always recorded in pathology reports (34, 45). Pathologists relied on the information provided by the surgeon when interpreting specimens which in turn affects the stage data provided in the pathology report (45). The development and adoption of synoptic structured reporting was recommended to ensure more consistent pathological information including cancer stage is provided to PBCRs (35, 45). Second, pathology reports provided substantial pathological information however often lacked the clinical information necessary for aspects of staging not related to the primary tumour (35, 43). Other data sources containing information on nodal involvement and distant metastasis were not as commonly available, including imaging reports and site-specific diagnostic and prognostic tests (35, 43).

#### 3.1.2. Processes and timing of data collection

This subtheme covers the PBCR data collection processes involved, as well as the timing of data collection used to determine population-based cancer stage at diagnosis.

Automated tools were used by some of the PBCRs to assist with cancer registration processes, such as the linkage of datasets, de-duplication of data, and consolidation of multiple notifications for a cancer case (11, 47). However, population-based cancer staging was often described as a manual process for many PBCRs, including those in England, New Zealand, and Australia (11, 34, 45, 47, 50). Manual processes included the review of data sources for specific data items, often the direct assignment of cancer stage if not explicitly recorded in data sources, and entry of data into cancer databases by cancer registry coders and officers (11, 34, 45, 47, 49). For New Zealand, manual data extraction and entry was performed by cancer registry coders who specialised in specific tumour types (45). Except for the use of some tools for linkage and de-duplication of data by the NCRAS, the rest of the cancer registration process involved the manual extraction of cancer information by cancer registration officers in England (47). Registry-Derived stage was manually assigned by Australian PBCRs during the national STaR project and required significant training and manual effort by cancer registry coders (11, 34). Degree of Spread (DoS), which reflected the extent of disease at diagnosis collected by the New South Wales (NSW) Cancer Registry on a population-based level, was provided through electronically coded hospital notifications however cancer registry coders also manually reviewed data sources (including pathology reports) to determine DoS (11). Training and detailed knowledge regarding cancer biology and staging were identified as key requirements for the efficient manual extraction of stage data and direct assigning of cancer stage (11, 34, 47, 49).

Though less commonly used, the benefits of computer algorithms for population-based cancer staging were recognised, including reducing the potential human error and variation associated with manual assignment of stage (40, 44). This was identified as the reason that Aitken et al. (40) programmed and utilised tumour-specific staging computer algorithms in their study on the feasibility of implementing the Toronto Childhood Cancer Stage Guidelines. Computer algorithm derived stage was checked against manually determined stage using the same staging criteria and reviewed when there was no agreement, with subsequent refinement of the algorithm and repeat testing until complete agreement was reached for multiple actual and hypothetical cases (40). Overall, there was good agreement between computer algorithm generated stage and manually assigned stage for most of the cancer types (40). However, the computer algorithms were only used in the step of calculating stage from data items manually extracted from medical records (40).

The Collaborative Stage (CS) System (also known as the Collaborative Stage Data Collection System) also utilised computer algorithms and was used by PBCRs in United States of America (USA) and Canada to collect stage data between 2004 and 2015 (42, 44, 49). The CS system was developed by the Collaborative Staging Task Force to enable collection of a specific set of data items that could be converted into multiple staging classification systems (44, 49). Manual extraction of tumour-specific data items and entry into the CS System fields was also required to enable computer algorithms to automatically generate the tumour stage. The algorithm subsequently generated the AJCC TNM 6th ed. summary stage group and SEER Summary Stages 1977 and 2000 for the cancer case (44). The CS has since been discontinued and was no longer routinely used (49). It was recognised that when the CS system was ceased in 2016, cancer registries had to transition from using the computer algorithm-based CS system to the manual assignment of cancer stage (49).

The timing of data collection was largely based on definitions of stage at diagnosis. Stage at diagnosis was defined as the stage derived within 120 days of the date of diagnosis and, as a result, 120 days was often used as the timespan for data collection to determine stage at diagnosis (11, 43). The NSW Cancer Registry used notification sources within 120 days of the date of diagnosis to determine tumour stage regardless of the staging classification system used (11). During the Australian STaR project, routine notification sources received within 120 days of the date of diagnosis were used to determine registry-derived stage (11, 34). The International Cancer Benchmarking Partnership publication by Cabasag et al. (43) included the recommendation that the timing of data collection for stage at diagnosis was prior to the date that primary treatment is initiated or within four months after the date of diagnosis, depending on whichever is earliest, to ensure comparable definitions of stage at diagnosis. However, it was unclear if four months was equivalent to 120 days exactly.

The exception to this related to the use of treatment procedure records available to the NCRAS, where records with treatment procedures within 30 days before or after the date of diagnosis were used to determine pathological stage (6). If there were multiple records within this data collection period, then the record with the date closest to the date of diagnosis was given priority over other records (6).

### 3.2. Theme 2: Staging classification systems

Multiple classification systems were developed for population-based cancer staging by different groups and for various purposes and cancer types. The staging classification systems were broadly grouped into three groups: tumour, node and metastasis based; categorised into localised, regional, and distant groups; and miscellaneous systems.

#### 3.2.1. Based on tumour, node, and metastasis

Staging classification systems with tumour, node, and metastasis stage variables were commonly used, including the AJCC TNM, condensed TNM, and essential TMN (7, 43, 48, 52). Included in this group were also two registry approaches to population-based cancer staging using AJCC TNM.

The AJCC TNM system is the most widely used cancer staging system in clinical practice for solid tumours (7, 11, 43, 52). It is maintained by the AJCC in collaboration with the Union for International Cancer Control (UICC), though the latter also has its own TNM system (known as UICC TNM) which is very similar to the AJCC version (7, 49).

AJCC TNM was based on either clinical information (cTNM) gathered prior to starting treatment (including physical examination, imaging, or endoscopy) or on pathological information (pTNM) (using microscopic examination of specimens) (5–7). However, whether TNM was either clinical or pathological was not always recorded (5). Potentially this represented integrated TNM stage based on both pathological and clinical data, for example from multidisciplinary team meetings (5). However, it may have also been used when it was unknown whether TNM was based on clinical or pathological evidence (5). The AJCC TNM staging guidelines are regularly revised based on current clinical evidence, resulting in differences between editions, however the edition used was not always recorded in data sources with the TNM stage (5, 6, 43).

Alphanumeric codes were assigned using tumour-specific rules to three stage variables based on size or depth of invasion of the primary tumour (for the T value), involvement of regional lymph nodes (*N* value), and presence of distant metastasis (*M* value) (5, 6, 11, 35, 43, 44, 49). Combining these values gave the overall TNM summary stage group ranging from I to IV (5, 43, 44). Some tumour types also required additional non-anatomical information to determine stage, such as the use of serum tumour markers for testicular cancer (11, 35). All three stage variables were required to determine TNM summary stage group, so when the TNM stage variables or other stage information was missing from data sources the TNM summary stage group could not be assigned (48, 49). To deal with this, a “non-restrictive approach” was used that assumed that missing and unknown T, N and M values were equivalent to a zero value, which allowed TNM summary stage group to be derived (5, 6, 35). Another approach utilised stage data from other staging classification systems to replace missing TNM information, resulting in a “reconstructed stage” (48).

The TNM system was the most used staging classification system by PBCRs (7, 43, 48, 52). It was used by 39% of European PBCRs in the 2013 study by Siesling et al. (52). Minicozzi et al. (48) reported that 25 of the 34 European PBCRs included in their 2017 study provided TNM stage data. TNM was additionally used by PBCRs in England, Ireland, Denmark, Norway, Sweden, Australia, and in USA and Canada when the CS system was utilised (5, 11, 34, 42–44, 47, 49). UICC TNM was the most preferred system for European PBCRs.

Condensed TNM was a simplified staging system developed by the European Network of Cancer Registries to allow PBCRs to determine TNM stage when any or all three TNM data elements are not recorded in data sources (7, 10). It was based on both clinical and pathological information, with preference given to pathology data for T and N (10). T was categorised based on tumour-specific definitions as either localised or advanced, and N and M as either absent or present (7, 10). The overall stage categories were tumour localised, tumour with local spread, tumour with regional spread, advanced cancer (including both metastatic and non-resectable tumours), or unknown extent (10). Compared to the TNM system, Condensed TNM was less commonly used (7, 48). For example, Minicozzi et al. (48) revealed that 56% of European PBCRs included in the study used Condensed TNM.

Essential TNM was a different simplified TNM staging system developed by the UICC and the IARC to allow stage to be derived when TNM stage data elements are not recorded (7). Site-specific staging guidelines using clinical and pathological information were presented in staging flowcharts and applied to breast, cervical, colorectal, and prostate cancers (7). T was classified as either localised or advanced, and both N and M were classified as either absent or present (with N further subdivided into regional limited and regional extensive if present) (7). The overall stage categories varied depending on the tumour type, ranging from localised limited, localised advanced, regional limited, regional extensive, and distant metastasis (7). These stage groups correlated with the TNM summary stage groups (7). It was unknown whether Essential TNM was used by any PBCRs however, the original article by Pineros et al. (7) reported pilot studies were occurring at the time of publication.

The two terms “Registry-Derived stage” and “registry defined stage” described two country-specific approaches to how the AJCC TNM system has been adapted for use by PBCRs for population-based cancer staging.

RD stage was the best estimate of TNM summary stage at time of diagnosis based on notification sources routinely available to PBCRs in Australia (11, 34). It was derived from the STaR project whereby stage was collected for the five highest incidence cancers (prostate, breast, lung, bowel, and melanoma) by jurisdictional PBCRs in Australia using simplified business rules based on the AJCC TNM 7th edition (11, 34). Cancer registry coders transcribed recorded T, N, and M values or manually assigned these values if missing, which required extensive training (11, 34). Overall, Lawrance et al. (11) found that RD stage had higher agreement with AJCC Stage Group compared to NSW's Degree of Spread. However, since the conclusion of the STaR project, it was no longer routinely collected.

Registry defined stage described the single anatomical stage at diagnosis assigned by NCRAS cancer registration officers based on the TNM system using all relevant information available, including multidisciplinary team meetings, pathology reports, imaging results, and autopsy reports (47). Due to the multiple data sources available to the NCRAS, inconsistencies may occur between different datasets for the same cancer case (5, 6). In response, a hierarchal approach that prioritised specific data sources for individual components of tumour stage was proposed (5, 6).

#### 3.2.2. Categorised into localised, regional, and distant

The second major category were population-based cancer staging systems that classified into localised, regional, and distant groups. This included Extent of Disease systems and the SEER Summary Stage system.

Extent of Disease (EoD) was a simplified staging system that classified into stage groups local, regional, and metastatic (5, 48). EoD was generally a locally developed system developed for population-based cancer staging by a specific PBCR (5). For example, the EoD staging system utilised by the NSW Cancer Registry was DoS, which was routinely collected for non-hematological malignancies (11). EoD was reportedly used by PBCRs in Norway, New Zealand, Finland, Austria, Portugal, Czech Republic, and Estonia (5, 43, 48, 53), with a number of other PBCRs in Europe that used it in combination with other staging classification systems (48).

The SEER Summary Stage 2018 was the most recent version of a SEER developed summary stage system used in USA (9). It applied to all cancer types and uses both clinical and pathological data (7, 9). There were six major stage groups (*in situ*, localised only, regional, distant site/node involved, benign/borderline, unknown if extension or metastasis) (9, 43). SEER Summary Stage was infrequently updated and often used for long-term analyses of cancer stage (7, 44, 45, 51). As it was a simplified staging system specifically developed for use by PBCRs, it was less complex to learn and use (7, 44). However, it was not directly comparable with TNM stage groups and outside of cancer registries it was not well known (7, 9). In addition to the PBCRs in USA, Canada and New Zealand PBCRs also reportedly used SEER Summary Stage (9, 43, 45).

#### 3.2.3. Miscellaneous staging classification systems

Miscellaneous staging classification systems did not fit into the two other groups, including the SEER Extent of Disease Coding system, the Toronto Childhood Cancer Stage Guidelines, and site-specific classification systems including FIGO and Duke's classifications. Generally, there was less information available on these staging classification systems and whether PBCRs currently use them.

SEER Extent of Disease Coding was a coding system used for cancer stage developed by SEER (39, 44, 51). It applied to all tumour types and combines clinical and pathological information (51). Coding rules were tumour-specific based on both SEER Summary Stage 2000 and AJCC TNM 8th ed (51). Stage was recorded as a ten-digit code which combined five different fields: the size of the primary tumour, extension of the tumour, lymph node involvement, the number of pathologically positive regional lymph nodes, and the number of regional lymph nodes that were pathologically assessed (44).

The Toronto Childhood Cancer Stage Guidelines (henceforth known as the Toronto Guidelines) were developed by an international panel of experts in collaboration with the UICC with the aim of creating the first globally consistent approach to the population-based cancer staging of childhood cancers (40, 41, 46). The Toronto Guidelines applied to the 16 most common paediatric cancer types, with both the staging rules and the overall stage groups varying based on tumour type (40, 41). Recognising the barriers regarding registry resources and data access, it used a two-tiered approach (40, 41, 46). Tier 1 criteria were less detailed and are intended for use by cancer registries with limited resources, such as those in low- and middle-income countries. Tier 2 criteria were more detailed and were developed for use by well-resourced cancer registries with access to data, like those in high-income countries. The Tier 2 categories could be collapsed down into the Tier 1 categories enabling comparisons (40, 41, 46). Aitken et al. (40) demonstrated the feasibility of implementing the Toronto Guidelines a within a PBCR in Australia, as an example of high-income country.

Though more often used in clinical practice, stage data from site-specific classification systems were collected by a few PBCRs (5, 7). FIGO is the acronym for the Fédération Internationale de Gynécologie et d'Obstétrique which developed the staging classification system for gynaecological cancers (7). Stage groups ranged from I to IV that are comparable to TNM summary stage groups, however was not easily converted into other staging systems (5). PBCRs in Norway reportedly collected FIGO stage data for ovarian cancer (5). Duke's classification was a staging system utilised for colorectal cancer though it was no longer commonly used (7). Stage groups ranged from Duke's A to D that could be converted into comparable TNM summary stage groups however could not be easily converted into other staging systems (5, 6). PBCRs in Norway and England reportedly collected Duke's stage for colorectal cancer (5, 6).

## 4. Discussion

This rapid scoping review highlighted the data sources used by PBCRs to collect population-based cancer stage and the various staging classifications used. Differences in how PBCRs collect stage data affects the comparability of stage data when making national and international comparisons between jurisdictions and countries (5–7).

Though the timing of data collection for cancer stage is largely based on the definition of cancer stage at diagnosis within 120 days, variability in the timespan of data collection for primary cancers has been noted across the literature (55). Research studies from France, the USA, and European countries examining the risk of secondary primary cancer utilised a period of two months to define the synchronicity period (the period following the date of the first cancer diagnosis which is excluded when evaluating the risk of a second primary cancer) (55). However, variability in the time interval utilised from one month to six months was recognised across different studies on secondary primary cancers (55).

The data sources used for population-based cancer staging depend on what data is routinely available for a PBCR's registration processes, which tends to be cancer notifications containing new cancer diagnoses. As cancer diagnoses generally require a pathological confirmation of diagnosis, pathology reports were one of the commonest data sources received by PBCRs. However, the limitations associated with pathology reports are well described, particularly regarding the completeness of stage recorded and limited clinical information (35, 43, 45). In relation to the first limitation, there is evidence supporting the role of structured pathology reporting in improving the use of standardised terminology and the completeness of cancer pathology reports, including the completeness of stage data (56–58). Recognising this, the International Collaboration on Cancer Reporting was formed in 2011 as an international collaboration of pathology organisations that develops internationally standardised and evidence-based pathology datasets for cancer reporting (56, 59). Improvements in the completeness of cancer stage reported on pathology reports would assist with the completeness of cancer stage collected by PBCRs from pathology reports and further research is required to explore this further. Regarding the second limitation, the data sources that may assist with determining these stage variables, such as imaging reports and MDT information, are not commonly available to PBCRs (35). Some PBCRs have utilised other approaches to deal with data gaps, such as the use of hospital notification data containing clinical codes for metastatic cancer (34). The major exception is the NCRAS which exemplifies a PBCR that does have access to the various data sources required to collect comprehensive stage data (47, 50). However, when multiple sources are used as seen with the NCRAS processes there is the risk of conflicting information, which is why a hierarchal approach to prioritisation of data sources for specific stage data items is recommended (6).

Like other cancer registration processes, population-based cancer staging remains a largely manual process for PBCRs (7, 45, 50). However, manual extraction of stage data requires significant effort and time to undertake, particularly when cancer stage recorded in data sources is incomplete or absent and needs to be manually assigned (7, 11, 49). Subsequently it was recognised that for PBCRs that have limited resources, the collection of stage data may not be justifiable (11). Computer algorithms, such as those used with the Toronto Childhood Cancer Stage Guidelines and the Collaborative Stage System, are examples of tools used to minimise the variation in manual interpretation and application of staging rules. Another such example is the CanStaging + tool which was the first internationally validated, open-source cancer staging tool developed based on ICBP data (60). Though these algorithms still rely on the manual extraction and input of specific data items.

Acknowledging the challenges associated with large volumes of manual work, artificial intelligence tools have been utilised within PBCRs to streamline the processing of medical documents such as automated case-finding (identification of new and existing cancer cases) and auto-coding (abstraction and coding of information into data fields) (61). Additionally, increasing emphasis has been placed on the development of automated tools to assist PBCRs with cancer staging and research has been conducted into artificial intelligence methods to extract cancer stage from pathology reports and medical records (54, 62, 63). It is unclear whether these tools are currently being used by PBCRs though it is expected that this field of research will continue to grow and may be utilised by PBCRs in the future.

Nationally driven initiatives and programs allow for large scale reforms and improvements that are well-resourced, enabling consistency between PBCRs in different jurisdictions and resulting in a tendency towards country-specific approaches to population-based cancer staging. Following the ICBP's investigations on why cancer survival was lower in the United Kingdom compared to other high-income countries, significant reforms were undertaken in England to standardise cancer registration practices (including the transition from eight regional cancer registries into a singular centralised registry and utilisation of more data sources for registry processes) establishing a comprehensive and consistent approach to registry staging practices in England (5, 64). The STaR project in Australia and the Collaborative Staging system in the USA are two other examples of national initiatives that enabled collection of consistent national-level stage data (11, 34, 44, 49). However, both were short-lived projects and are no longer routinely used. Such temporal changes in population-based cancer staging have implications for comparability, as the stage data collected by one method for specific period is not necessarily comparable to data collected subsequently by other approaches. In the absence of national-level prioritisation, PBCRs in different jurisdictions may lack a consistent and comparable approach to population-based cancer staging, such as using different or multiple staging classification systems (48, 52). Though the use of multiple different staging classification systems by a PBCR may be intentional to manage data gaps and issues with completeness of stage data (48). Of the 62 European PBCRs examined by Minicozzi et al. (48), 24 collected stage data using two or more of TNM, EoD, and condensed TNM staging classification systems. Considering this, there would be value in the development of an international tiered framework for the collection of staging that considers data sources, resources, collection and validation, and outputs. Appropriate detail should be included to facilitate the application of the framework at the jurisdictional level, and provide relevant context to the interpretation of staging data when undertaking benchmarking and comparative analysis.

Despite being the most common staging classification, the complex staging criteria of the AJCC/UICC TNM system poses well-described challenges to the collection of complete stage data by PBCRs and has driven the development of the various simplified staging systems (7, 11, 49). The use of different staging classification systems presents a major barrier to the comparability of stage data (5, 7, 43, 45, 53). To allow for comparisons, staging conversion algorithms have been developed and utilised to convert stage data from one staging classification system into another. Whilst more challenging to collect, individual TNM stage values capture highly detailed tumour information that can be more easily converted into other staging classification systems, through mapping of individual T, N and M categories against stage categories of other staging classification systems (5). Articles have developed and used algorithms to convert from AJCC T, N and M categories into Condensed TNM (48), SEER Summary Stage 2000 [as a surrogate for EoD stage groups (5, 43)], and EoD (53) for specific tumour types. Combining the individual TNM categories loses the more granular stage information, making it harder for AJCC TNM summary stage groups to be converted into other staging classification systems (5). The opposite conversion from less detailed simplified staging systems to more detailed staging systems cannot easily be done without the addition of supplementary stage information (5, 53). As EoD captured less detailed tumour information, there were difficulties in converting to other more detailed staging systems (like TNM) in the absence of supplementary stage information (5, 7, 43, 45, 53). Though the CS system mapped the localised, regional, and distant groups of SEER Summary Stage with TNM stage groups, the algorithms required additional site-specific factors (such as tumour grade, hormone receptor status, and treatment response) that were not captured by EoD (5, 53). As a result, other PBCRs have not been able to use the same algorithms to convert from EoD because they lack complete dataset collected by the CS system, which consists of data items required to determine both the AJCC TNM and SEER Summary Stage (5, 53). Regardless of the staging systems involved, using stage conversion algorithms is associated with misclassification risks, which generally varies by tumour type and according to how well stage groups map to one another (5).

There was a lack of consistent terminology regarding cancer stage data collected for population-based analyses. This has consequences in not knowing if the similar terms have the same meaning or something else entirely (65). “Population-based cancer stage” is a term that has been used in the literature (35, 43), however it is not utilised universally. “Stage at diagnosis” is another commonly used term (5, 6, 34, 46, 48, 52) however without addition of terms like “population-based” or “population-level”, it can be unclear if this refers to the collection of individual-level stage at diagnosis. Two further terms that were outlined were “registry-derived stage” and “registry defined stage.” Due to its purpose to capture consistent population-level stage data across Australian PBCRs, RD stage is clearly defined as the “best estimate of summary TNM stage at time of diagnosis as derived by cancer registries from available data sources for use in population data analysis” (12). Since the project's conclusion, RD stage has been used to refer to the specific datasets collected during the STaR project (11, 34) however it is not a term that is used outside of Australia (11). Registry defined stage is a similar term used by the NCRAS however it is not as commonly used or as clearly defined as RD stage (47). Due to the number of staging classification systems that exist, the name of the specific staging classification system may be used instead. However, the interpretation of terminology may be complicated as similar terms may be used in different contexts. For example, “extent of disease” is used to describe the anatomical extent of cancer growth and spread (7, 10) in addition to its use to describe the locally developed Extent of Disease staging systems (5, 43, 48, 53) and SEER's Extent of Disease coding system (51). Furthermore, some articles utilise four months as the period of data collection for population-based cancer stage whereas others use 120 days. Given the potential difference in number of days depending on the specific month, there is a need to clarify whether four months is equivalent to 120 days, based on a standard month of 30 days duration. A concept analysis is recommended to further review the clarity of terms and provide a clear definition for population-based cancer stage at diagnosis (65–67).

## 5. Strengths and limitations

This paper is the first published review looking into this area at the time of the literature search and there were relatively few articles included in the analyses, demonstrating a lack of literature in this field generally. Another strength was the use of comprehensive search strategy inclusive of peer-reviewed articles and grey literature. Additionally, this review provided a comprehensive summary of the approaches used for population-based cancer registry staging which will inform the development and implementation of population-based cancer staging into the WACR.

A rapid scoping review was conducted due to time constraints which resulted in simplification of processes. The identification of literature and data extraction was conducted by a single reviewer however a second reviewer assisted with screening a selection of articles by title and abstract and reviewed articles queried with the full-text review. The search for grey literature from websites was limited to a few websites and particularly for larger countries with established PBCRs and organisations with international collaborations, which risks missing literature from smaller countries and less-well established PBCRs. Grey literature was also limited to documents that are publicly available, whereas it is expected that a large amount of information on PBCR processes is not available publicly and stored internally. The inclusion of articles only in English potentially excluded evidence from eligible studies in other languages.

## 6. Conclusion

PBCRs used different approaches to determine cancer stage at diagnosis at a population-level. Variation exists between PBCRs in what data sources were available, how they used them, and what staging classification system was used. The lack of an internationally standardised methodology for determining population-based stage at diagnosis hinders the ability to make comparisons between different jurisdictions and countries. However, the approaches used by PBCRs to collect population-based cancer stage are limited by the resources available and the data sources that are accessible. This rapid scoping review adds to the limited literature that currently exists on the approaches used by PBCRs to determine cancer stage at diagnosis on a population-level. Additionally, it provides comprehensive background information on the collection of population-based cancer stage that will inform current work to integrate such processes into the WACR. Development of international guidelines, for example the development of a tiered framework for the collection of staging, would assist PBCRs with consistent collection of population-level stage at diagnosis and improve comparability of stage data, though consideration should be given to the significant amount of manual work that is required to collect population-based cancer stage.
